# Preclinical evaluation of waste-derived pomegranate extract (PWE) as a potential preventing and therapeutic agent for benign prostatic hyperplasia

**DOI:** 10.3389/fmolb.2026.1769028

**Published:** 2026-02-19

**Authors:** Valeria Consoli, Agata Grazia D’Amico, Claudio Russo, Emanuele Foderà, Daniela Passarella, Antonio Pecorino, Velia D’Agata, Luca Vanella, Valeria Sorrenti

**Affiliations:** 1 Department of Drug and Health Sciences, University of Catania, Catania, Italy; 2 Department of Drug and Health Sciences, CERNUT-Interdepartmental Research Centre on Nutraceuticals and Health Products, University of Catania, Catania, Italy; 3 Department of Medicine and Health Sciences “V. Tiberio”, University of Molise, Campobasso, Italy; 4 Mediterranean Nutraceutical Extracts (Medinutrex), Catania, Italy; 5 Department of Biomedical and Biotechnological Sciences, University of Catania, Catania, Italy

**Keywords:** agricultural wastes, BPH, circular economy, inflammation, nutraceuticals, oxidative stress

## Abstract

**Introduction:**

Benign prostatic hyperplasia (BPH) is a highly prevalent age-related condition, affecting nearly half of men over 60 and up to 80% over 80 years old. Chronic inflammation and oxidative stress are recognized contributors to BPH progression and may facilitate the development of prostate cancer. Growing interest in sustainable, natural chemopreventive agents has highlighted agro-industrial by-products as valuable sources of bioactive compounds.

**Methods:**

In this study, we evaluated the protective and therapeutic effects of pomegranate waste extract (PWE), a phytochemical-rich by-product of Mediterranean agri-food processing, in a testosterone-induced rat model of BPH.

**Results and Discussion:**

Immunohistochemistry and Western blot analyses revealed that testosterone administration reduced the expression of PECAM-1 and increased the levels of NRF2, HO-1, and IL1R1, consistently with endothelial dysfunction, oxidative stress, and inflammation. Co-treatment with PWE restored these markers toward control levels, indicating attenuation of testosterone-driven molecular alterations. Proteomic profiling further demonstrated that testosterone dysregulated proteins involved in mitochondrial function, redox balance, and DNA repair; on the contrary, PWE normalized their levels and enhanced antioxidant enzymes. In periprostatic adipose tissue, PWE counteracted testosterone-induced upregulation of HO-1, NRF2, and GPX4. Overall, PWE mitigates oxidative, inflammatory, and metabolic dysfunctions associated with experimental BPH, supporting its potential as a sustainable natural chemopreventive strategy.

## 1 Introduction

Benign prostatic hyperplasia (BPH) is a highly prevalent, age-associated urological condition in older men, with a steadily increasing global burden. In 2019, the estimated number of cases reached 94 million worldwide, affecting approximately half of men in their sixth decade of life and up to 80% of those in their ninth decade (Collaborators, 2022). Alongside BPH, prostate cancer (PCa) represents the most frequently diagnosed malignancy in men and, according to GLOBOCAN 2020, remains the second leading cause of cancer-related mortality after lung cancer (Stewart and Lephart, 2023; Ahmed et al., 2022).

BPH manifests as the non-malignant proliferation of stromal and epithelial cells predominantly within the prostate’s transitional zone (TZ), leading to glandular nodular enlargement that compresses the urethra and precipitates lower urinary tract symptoms (LUTS), including nocturia, weak stream, and incomplete emptying. Pathogenetically, the process is initiated by systemic testosterone, which diffuses into prostate stromal cells and undergoes irreversible conversion to dihydrotestosterone (DHT) by type II 5α-reductase, the predominant isoform in prostatic tissue. Exhibiting 2- to 10-fold greater affinity for the androgen receptor (AR) relative to testosterone, DHT induces AR phosphorylation, nuclear translocation, and dimerization, thereby activating a transcriptional cascade that governs prostatic cellular homeostasis. Moreover, accumulating evidence indicates that inflammatory and oxidative stress-related pathways actively contribute to cellular proliferation in BPH. Reactive oxygen species are known to participate in the early phases of carcinogenesis, and prostatic hyperplasia is increasingly regarded as a premalignant condition with the potential to progress toward prostate cancer, although experimental and clinical findings on this relationship remain heterogeneous and, in some cases, controversial (Roehrborn, 2008). Although not malignant in nature, untreated BPH may contribute to the onset of PCa, a disease predicted to affect one in six men during their lifetime (Collaborators, 2022; Suburu and Chen, 2012). Evidence shows that cells derived from BPH, unlike those from healthy prostatic tissue, are capable of stimulating epithelial growth *in vivo*, thereby supporting a link between BPH and PCa (Buhigas et al., 2022). Consistently, numerous studies indicate that chronic inflammation plays a central role in both disorders (Ørsted and Bojesen, 2013; Alcaraz et al., 2009). Inflammatory infiltrates not only promote the initial development of BPH and PCa but may also contribute to tumor progression and metastasis formation (Baci et al., 2019).

**FIGURE 1:**
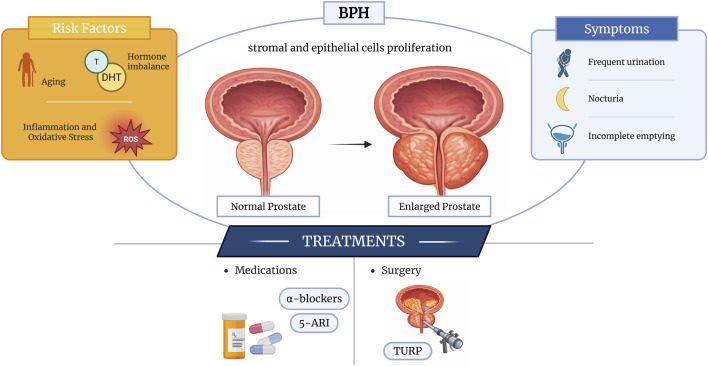
Schematic representation of BPH pathology and current treatments.

Transurethral resection of the prostate (TURP) remains the reference surgical intervention for the management of BPH; however, its long-term clinical benefit is debated due to the occurrence of postoperative complications and symptom recurrence. Current pharmacological management primarily relies on α-adrenergic antagonists (e.g., doxazosin and tamsulosin) and 5-α-reductase inhibitors (e.g., finasteride and dutasteride), which are effective in alleviating lower urinary tract symptoms and reducing the risk of disease progression (Bae et al., 2016; Parsons et al., 2020). Nonetheless, these therapies are frequently limited by adverse effects, including hypertension, erectile dysfunction, reduced libido, and other drug-related complications, which can compromise patient adherence and long-term treatment efficacy (Zaman Huri et al., 2014) (Figure 1). Thus, increasing attention has been directed toward alternative and complementary approaches to disease prevention and management. Natural bioactive compounds, including polyphenols, flavonoids, carotenoids and phytosterols are recognized for their chemopreventive potential in prostate cancer and management of BPH (Stefanucci et al., 2024). These molecules are abundantly found in plant-based foods but are also recoverable from agro-industrial by-products. Within the framework of environmental sustainability and the circular economy, the valorization of agricultural waste as a source of high-value bioactive compounds has gained significant relevance. Such compounds can be repurposed across multiple sectors, including food, nutraceuticals, cosmetics, and even packaging (Bekavac et al., 2025; Reguengo et al., 2022; Saini et al., 2025).

Based on this rationale, the present study aimed to investigate the chemopreventive and therapeutic properties of extracts derived from Mediterranean agri-food waste, specifically pomegranate by-products, using an *in vivo* model of BPH.

## 2 Materials and methods

### 2.1 Animals

All animal experimental procedures were performed on six-weeks-old male (150 g) RccHan:WIST rats (Envigo Laboratories, Indianapolis, IN, United States). Rats were housed in three animals per cage under a 12/12 h light/dark cycle at a constant temperature (23 °C–25 °C) with free access to food and water and were allowed to acclimate for at least 1 week after arrival before starting all experiments, which were conducted between 9:00 a.m. and 3:00 p.m. This study was executed according to the European Communities Council directive and Italian regulation (EEC Council 2010/63/EU and Italian D.Lgs. No. 26/2014) to replace, reduce, and refine the use of laboratory animals. All procedures were approved by the ethical committee of the University of Catania (OPBA) and by the Italian Ministry of Health (authorization no. 231/2023-PR). Animals were divided into three experimental groups: Control group (sham operation group), BPH group and BPH orally administered with pomegranate extract (PWE). Rats of the BPH group were injected IP (Intraperitoneal inoculation) with testosterone propionate (10 mg/kg) in corn oil, every 2 days for 2 weeks, in line with literature reports (Paterniti et al., 2018). Rats of the BPH model group were orally administrated with or without PWE as aqueous solution at 0.6%, added with 1% sucrose, which was replenished every 2 days. The commercial dry powdered pomegranate extract (PWE; Medgranate™, Medinutrex, Catania, Italy) used in this study was derived from “Wonderful” variety pomegranate by-products (exhausted peels, membranes, and arils) via industrial drying to 5% moisture, hydroalcoholic extraction (ethanol/water 60:40 v/v, 1:10 w/v, 24 h stirring), alcohol recovery, and spray-drying (∼30% yield), and has been previously characterized and validated for its polyphenolic profile and bioactivity in *in vitro models* of prostate cell lines demonstrating antioxidant and anti-proliferative effects (Consoli et al., 2023). At the end of the experiment, animals were euthanized by CO_2_ inhalation with a flow rate of about 50% (5 L/min) and prostate, liver and periprostatic adipose tissue (PPAT) were excised. Prostate tissue was used for histological, immunohistochemical, and biochemical analyses, liver and PPAT tissues were processed for proteomic and biochemical analyses.

### 2.2 Hematoxylin and eosin (H&E) staining and histological analysis

Rat prostate tissues were immediately fixed in 10% formalin for 24 h and were then paraffin-embedded and sectioned at a thickness of 5-μm. The sections were processed for hematoxylin-eosin (H&E) staying as previously described (Ginja et al., 2019). Briefly, selected sections were deparaffinized in xylene and rehydrated in alcohol concentrations. Subsequently, sections were stained with hematoxylin and eosin (H&E; Sigma-Aldrich, Egypt) for histological examination.

### 2.3 Immunohistochemistry (IHC) analysis

The expression and distribution of Platelet endothelial cell adhesion molecule (PECAM-1), Hemeoxygenase-1 (HO-1), and Nuclear factor erythroid 2-related factor 2 (Nfr2) in rat prostate tissue was evaluated through immunohistochemical analysis as previously described (D’Amico et al., 2013; D’Amico et al., 2023). Briefly, the sections were incubated overnight at 4 °C with the specific primary antibodies, and then incubated with 3,3′-diaminobenzidine solution (DAB substrate Kit; SK-4100, Vector Laboratories, Burlingame, CA, United States). The immunoreaction was visualized with an Axioplan Zeiss light microscope (Carl Zeiss), and the micropictures were captured using a digital camera (AxioCam MRc5, Carl Zeiss) via AxioVision Release 4.8.2—SP2 Software (Carl Zeiss Microscopy GmbH, Jena, Germany) (scale bar 50 μm).

### 2.4 Proteomic analysis by LC-MS/MS

For proteomic analysis, Filter-Aided Sample Preparation (FASP) of liver and prostate homogenates was performed by using an adapted protocol of Sielaff et al.(2017). Briefly, 40 μg of proteins were transferred onto a Nanosep 10-kDa-cutoff filter (Pall Corporation, Portage, MI, United States) previously rinsed with 100 μL of 1% (v/v) formic acid (FA), the filter was washed two times with 200 μL urea buffer (8 M urea and 100 mM Tris, pH 8.5 in Milli-Q water) to remove the detergents present in the extraction buffer. 100 μL of dithiothreitol (DTT) solution (8 mM DTT in urea buffer) was added to the filter unit, covered with tin foil, and incubated for 15 min at 56 °C in a dry block heating system to reduce proteins. After two washes with 100 μL of urea buffer, the proteins were alkylated by adding 100 μL of 2-iodoacetamide (IAA) solution (50 mM IAA in urea buffer) and incubating in the dark for 20 min at room temperature. 100 μL of ammonium bicarbonate (AB) buffer (50 mM in milliQ water) was added to the filter for the buffer exchange. The digestion was performed with trypsin: a trypsin stock solution (1 μg/μL) was prepared, dissolving the enzyme in 50 mM acetic acid; 50 μL of trypsin working solution (0.01 μg/μL in AB buffer) was added onto the filter and incubated overnight at 37 °C in a wet chamber. The peptide mixture was collected by centrifugation and acidified with 10% (v/v) trifluoroacetic acid (TFA) to a final TFA concentration of 0.2% (v/v). The peptides obtained with the FASP protein digestion were analyzed on a nanoflow liquid chromatography (nLC) system (Ultimate 3000 UHPLC) coupled to an Orbitrap Fusion mass spectrometer (Thermo Fisher Scientific, Waltham, MA, United States), operating in positive ionization mode with spray voltage set at 1.7 kV and source temperature at 275 °C, equipped with a nanoEASY-Spray ion source. The solvents used for the chromatographic system were: solvent A, consisting of 100% water with 0.1% formic acid, and solvent B, consisting of 80% acetonitrile with 0.1% formic acid. Samples were loaded on a PepMap100 C18 pre-column cartridge (5 µm particle size, 100 Å pore size, 300 µm i.d. × 5 mm length, Thermo Fisher Scientific, Waltham, MA, United States) and separated on an EASY-Spray PepMap RSLC C18 column (75 μm i.d., 3 μm particle size, 100 Å pore size, Thermo Fisher Scientific, Waltham, MA, United States) at a flow rate of 300 nL/min and a temperature of 40 °C. The gradient was as follows: from 5% to 15% B in 20 min, from 15% to 35% B in 20 min, from 35% to 50% B in 5 min, and from 50% to 90% B in 2 min, with total LC runtime of 60 min. Data were acquired with Xcalibur software v4.4 (Thermo Fisher Scientific, Waltham, MA, United States). Full mass scans (MS1) were recorded in the Orbitrap analyzer at a resolving power of 120,000 (at *m/z* 200), with a mass range of *m/z* 350–1,500, automatic gain control (AGC) target of 4.0 × 10^5^, and a maximum injection time of 50 ms. Data-dependent MS/MS (MS2) analysis was conducted in top speed mode with a 1-second cycle time, where the most abundant multiple-charged (^2+^-^5+^) precursor ions were selected for activation in order of abundance and detected in linear ion trap at rapid scan rate. Quadrupole isolation with a 1.6 *m/z* isolation window was used, and dynamic exclusion was enabled for 30 s after a single scan. For MS2, automatic gain control was 1.0 × 10^3^, and the maximum injection time was 35 ms. The fragmentation mode was the Higher-energy collisional dissociation (HCD) with 30% normalized collision energy. The raw data were processed using Proteome Discoverer version 2.5 (Thermo Fisher Scientific, Waltham, MA, United States) with the MS1 Precursor option. The post-translational modification (PTM) profile was set as follows: fixed cysteine carbamidomethylation (ΔMass: 57.02), variable methionine oxidation (ΔMass: 15.99). Non-specific cleavage was allowed to one end of the peptides, with a maximum of 2 missed cleavages and Trypsin enzyme specificity. The highest error mass tolerances for precursors and fragments were set at 10 ppm.

For quantification, Minora Feature Detector node was employed to detect chromatographic peaks, while Precursor Ion Quantifier node was used for precursor quantification, applying Unique peptides only. Normalization was performed based on the Total Peptide Amount. Protein abundance was calculated using the Top N Average method (N = 3), protein ratios were determined using a Pairwise ratio–based approach, and statistical significance was assessed using a Background-based t-test. Spectra were matched against the *Rattus norvegicus* (TaxID = 10,116) database downloaded from the Uniprot website (8,205 entries). A false discovery rate (FDR) threshold of 1% was applied at the protein level.

### 2.5 Western blot analysis

Western blot analysis was conducted to evaluate PECAM-1, NRF-2, HO-1, GPX4 and IL1R1 expression levels on prostatic tissue and PPAT. Samples were processed using RIPA buffer (Thermo Fisher Scientific, Paisley, United Kindom) in the presence of phosphatase and protease inhibitors (Thermo Fisher Scientific, Paisley, United Kindom) for protein extraction. Subsequently, each sample was homogenized and sonicated twice for 20 s using an ultrasonic probe (Vibra cell, Sonics, Newtown, United States). The protein samples (70 μg) were diluted in 2× Laemmli sample buffer (Bio Rad, Berkeley, CA, United States) and heated at 85 °C for 5 min. Proteins were separated via electrophoresis and then transferred as previously reported by Ciaffaglione et al. (2022). Membranes were incubated overnight with HO-1 (GTX101147, diluted 1:1000, GeneTex, Irvine, CA, United States), NRF-2 (ab62352, diluted 1:1000, Abcam), PECAM-1 (SAB5700639, Sigma Aldrich, St. Louis, MO, United States), GPX4 (ab231174, diluted 1:1000, Abcam) and β-actin (GTX109639, diluted 1:7000, GeneTex) primary antibodies. Appropriate secondary antibodies were used to detect blots (diluted 1:10000). Blots were scanned, and densitometric analysis was performed with the Odyssey Infrared Imaging System (LI-COR, Milan, Italy). Values were normalized to β-actin.

### 2.6 Thiol groups determination

Concentration of non-protein thiol groups (RSH), reflecting about 90% of GSH cellular content, was measured in prostate tissue homogenates. RSH levels were evaluated by a spectrophotometric assay based on the reaction of thiol groups with 2,2-dithio-bis-nitrobenzoic acid (DTNB). DTNB solution and samples were mixed and incubated at room temperature for 20 min in the dark until a noticeable appearance of a yellow color was observed. After incubation, samples were centrifugated at 3,000 rpm for 10 min. The supernatant was collected and set in a black 96-well plate for measurement of absorbance in a microplate reader (Biotek Synergy-HT, Winooski, VT, United States) at λ = 412 nm. Experiments were conducted in quadruplicate. Results are expressed as pmoles/µL.

### 2.7 Measurement of lipid peroxidation

Levels of LOOH were evaluated through the oxidation of Fe^2+^ to Fe^3+^ in the presence of xylenol orange. Assay mixture consisted of 200 µg of sample (tissues homogenates), 100 µM xylenol orange, 250 µM ammonium ferrous sulphate, 90% ethanol, 4 mM butylated hydroxytoluene and 25 mM H_2_SO_4_. Samples were incubated at room temperature for 30 min and finally the absorbance was measured at λ = 560 nm using a microplate reader (Biotek Synergy-HT, Winooski, VT, United States). Calibration was obtained using hydrogen peroxide (0.2–20 µM). Results were expressed as fold change.

### 2.8 Cytokines levels quantification by enzyme-linked immunosorbent assays (ELISA)

IL-10 levels were assessed on prostate tissues homogenates using enzyme-linked immunosorbent assays (ELISA) (RBELR-IL10-CL-1, RayBiotech, Norcross, GA, United States) according to the manufacturer’s instructions. The absorbance was measured using a microplate reader (Biotek Synergy-HT, Winooski, VT, United States). The same technique was used for Adiponectin and Leptin levels quantification on PPAT homogenates (JXE0758Ra-96, JXE0561Ra-96 Shanghai, Cina). The results are expressed as pg/mL for IL-10, ng/mg prot for Adiponectin, and pg/mg prot for Leptin.

### 2.9 Proteomic data visualization and trend analysis

Protein abundance ratios between groups were calculated from label-free quantification values and log_2_-transformed prior to visualization. Heatmaps were generated to display relative abundance patterns across conditions, including proteins that did not reach statistical significance in differential analysis. These non-significant changes are presented for descriptive and hypothesis-generating purposes only and were not interpreted as confirmed effects.

### 2.10 Statistical analysis

The statistical analyses were performed using the software Prism v. 8.0.1 (GraphPad Software, San Diego, CA, United States) and Microsoft Excel v. 16.54. At least three independent experiments were performed for each analysis. Statistical significance (p < 0.05) of the differences between the experimental groups was determined by Fisher’s method for analysis of multiple comparisons. For comparison between treatment groups, the null hypothesis was tested by either a single-factor analysis of variance (ANOVA) for multiple groups or an unpaired t-test for two groups, and the data are presented as mean ± SEM.

## 3 Results

### 3.1 Effect of treatments on animal body weight and morphological changes in prostate tissues

**FIGURE 2:**
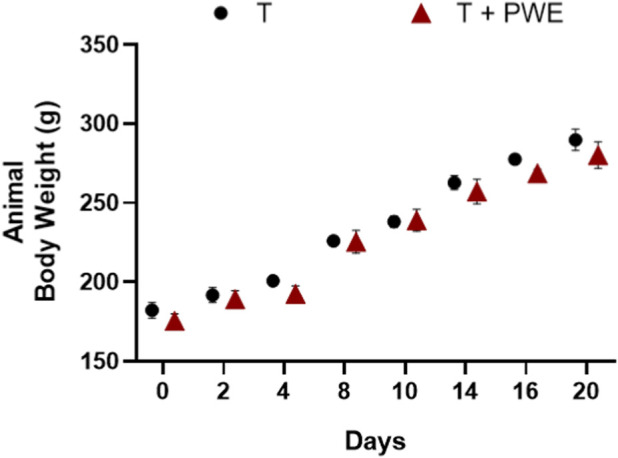
Animal body weight variations throughout T and T + PWE administration.

Body weight was monitored throughout the entire treatment period, and no significant differences were observed among the experimental groups (Figure 2). This finding suggests that the treatments did not exert adverse systemic effects, thereby supporting the reliability of the subsequent analyses focused on prostate-specific outcomes. Indeed, the prostate did not exhibit a significant increase in volume, most likely reflecting an early stage of benign prostatic hyperplasia (BPH), in which only mild clinical symptoms are typically observed and substantial prostatic enlargement has not yet occurred.

### 3.2 Morphological changes in prostate tissues

**FIGURE 3:**
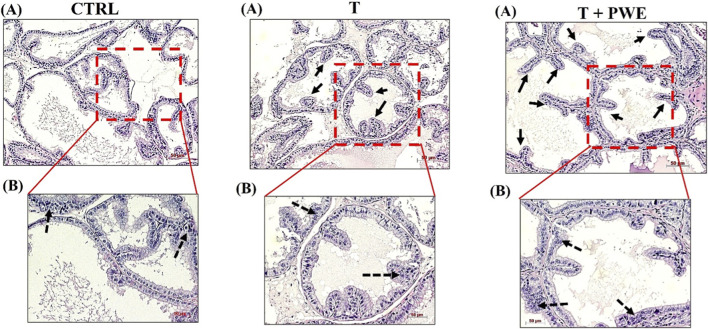
Representative photomicrographs from rat tissue sections of ventral prostate stained with hematoxylin and eosin. The digital micrographs [**(A,B)** ×10 and ×20 magnification, respectively] represent randomly selected fields. These images were acquired using the Zeiss Axioplan light microscope (Carl Zeiss, Oberkochen, Germany) with a digital camera (AxioCam MRc5; Carl Zeiss). Scale bar: 50 μm.

**FIGURE 4:**
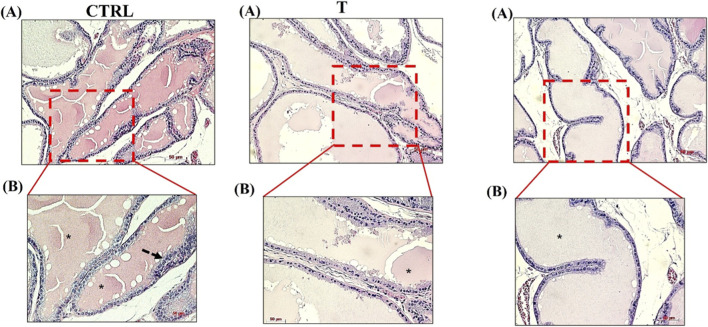
Representative photomicrographs from rat tissue sections of lateral prostate stained with hematoxylin and eosin. The digital micrographs [**(A,B)** ×10 and ×20 magnification, respectively] represent randomly selected fields. These images were acquired using the Zeiss Axioplan light microscope (Carl Zeiss, Oberkochen, Germany) with a digital camera (AxioCam MRc5; Carl Zeiss). Scale bar: 50 μm.

The morphological examination based on H&E staining showed a prostate histoarchitecture in the control group (CTRL) or following testosterone (T) and PWE treatment (T + PWE). Figures 3, 4 show sections of the ventral prostate (A and B, ×10 and ×20 magnification, respectively). In the control group, the acini showed regular sizes and shapes with epithelium consisting of a single layer of cuboidal or columnar cells or a pseudostratified columnar epithelium resting on the basal lamina and with few villous projections. The cells’ nuclei are basally located. Testosterone (T) and PWE groups are characterized by columnar cells with basal nuclei and perinuclear cytoplasmic clear areas, villous projections (thick arrows) more in PWE than in testosterone, and pseudostratified columnar epithelium lining (dashed arrows).

### 3.3 Oxidative stress and inflammation in rat prostate tissue

To verify whether the histological alterations observed were associated with oxidative stress and inflammatory processes, the immunoreactivity and distribution of key proteins involved in these pathways, specifically PECAM-1, NRF2, and HO-1, were evaluated.

**FIGURE 5:**
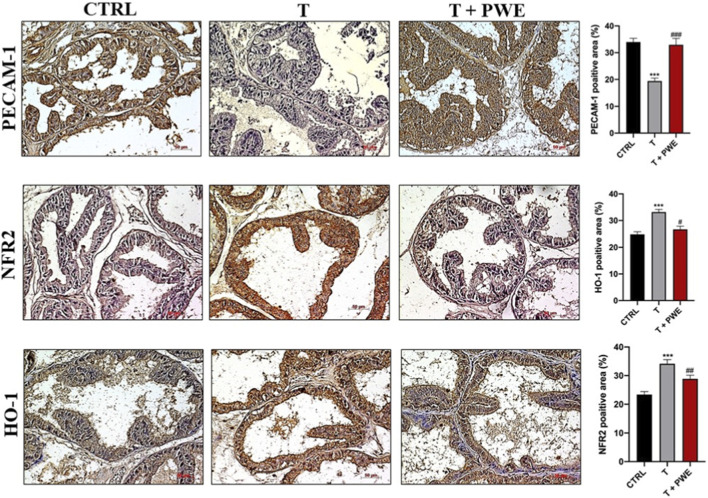
Immunodetection of PECAM-1, NFR2, and HO-1 in rat ventral prostate sections following testosterone (T) and PWE treatment. The micropictures were acquired by using Zeiss Axioplan light microscope (Carl Zeiss, Obrkochen, Germany) equipped with a digital camera (AcioCam MRc5; Carl Zeiss). The representative images are randomly selected and captured at ×20 magnification (scale bar 50 μm). The bar graph shows the PECAM-1, NFR2, and HO-1 positive area percentage (%) resulting from IHC analysis and reporting data mean ± SEM (^***^p < 0.001 vs. CTRL, ^#^p < 0.05, ^##^p < 0.01 or ^###^<p < 0.001 vs. T).

**FIGURE 6:**
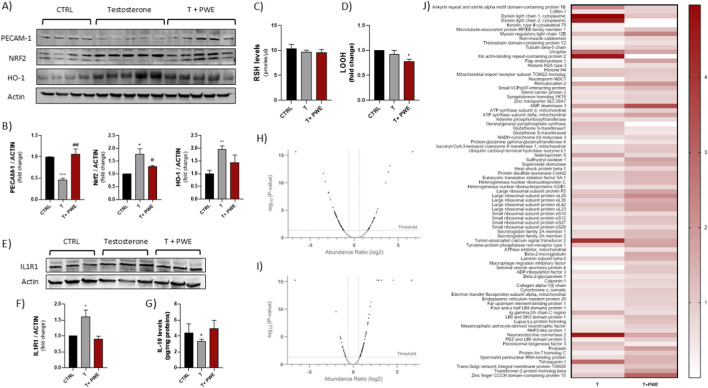
**(A,B)** Representative images and quantification of PECAM-1, Nrf2, and HO-1 protein expression by Western blot. **(C)** Levels of total thiol groups (RSH) and **(D)** lipid hydroperoxides (LOOH) in prostatic tissue homogenates. **(E,F)** Expression analysis of IL1R1 and **(G)** quantification of IL-10 levels. **(H,I)** Volcano plot depicting differentially expressed proteins in prostate tissue from respectively testosterone-treated (T) vs. control (Ctrl) groups and (T + PWE) vs. (T). Each point represents a protein, with x-axis indicating log_2_ abundance ratio and y-axis showing -log_10_ adjusted p-value. Horizontal dashed line denotes significance threshold (adj. p < 0.05); vertical dashed lines mark fold-change thresholds (±1.5). Proteins significantly downregulated (blue), upregulated (red), or unchanged (grey) are color-coded. **(J)** Representative proteomic heatmap of differentially expressed proteins following treatments. Heatmap illustrating the abundance ratios (0–4) of proteins modulated in rat prostatic tissue after treatment with T or T + PWE. Rows correspond to individual proteins identified by LC-MS/MS analysis, and columns represent the treatment conditions. Color intensity reflects normalized protein abundance, with darker shades indicating higher expression levels. Results are expressed as mean ± SEM (^***^p < 0.001, ^**^p < 0.001, ^*^p < 0.01 vs. CTRL, ^#^p < 0.05, ^##^p < 0.01 vs. T).

Immunodetection of PECAM-1, NRF2, and HO-1 in ventral prostate sections showed significant molecular alterations following testosterone administration and a modulatory effect induced by co-treatment with PWE (Figure 5). Testosterone exposure led to a pronounced reduction in PECAM-1 expression, consistent with endothelial impairment and early vascular remodeling events associated with early prostatic hyperplasia. In contrast, NRF2 and HO-1 levels were markedly upregulated in T-treated prostates, indicating activation of antioxidant and cytoprotective pathways in response to androgen-induced oxidative stress. Remarkably, co-treatment with PWE restored PECAM-1, NRF2, and HO-1 expression toward control levels, reflecting the extract’s capacity to mitigate testosterone-driven oxidative imbalance and preserve redox homeostasis within prostatic tissue (Figure 5). Western blot analysis further supported the immunohistochemical observations, confirming the modulatory effects of PWE co-treatment on testosterone-induced alterations. Quantitative protein evaluation revealed a significant decrease in PECAM-1 expression following T administration, consistent with endothelial dysfunction and vascular alteration within the prostatic microenvironment. Conversely, NRF2 and HO-1 protein levels were increased in T-treated groups, reflecting the activation of compensatory antioxidant defenses in response to oxidative stress. Importantly, co-treatment with PWE effectively normalized the expression of all three markers, restoring PECAM-1, NRF2, and HO-1 protein levels to values comparable to controls (Figures 6A,B). Analysis of redox biomarkers revealed that intracellular levels of reduced glutathione (GSH), measured as total thiol groups (RSH), remained unaltered across all experimental groups, indicating that neither testosterone administration nor PWE treatment significantly affected the cellular thiol antioxidant pool (Figure 6C). Conversely, lipid hydroperoxide (LOOH) levels exhibited a slight decrease in the PWE co-treatment group, reaching values marginally below those observed in control group (Figure 6D). This mild reduction suggests that PWE may exert a modest protective effect against lipid peroxidation, contributing to the maintenance of membrane integrity and redox homeostasis under testosterone-induced oxidative conditions. Moreover, IL1R1 expression was also increased upon T treatment, suggesting enhanced inflammatory signaling within the prostatic tissue, while PWE co-treatment restored protein levels similarly to untreated group (Figure 6F). In addition, IL-10 levels measured by ELISA revealed a slight but significant reduction after testosterone administration, consistent with a shift toward a pro-inflammatory microenvironment (Figure 6G). Notably, no alterations in IL-10 secretion were observed in the T + PWE group compared to controls, indicating that PWE co-treatment prevented the testosterone-induced decline of this anti-inflammatory cytokine.

### 3.4 Proteomic analysis in prostate tissues

Results from volcano plot analysis showed that comparing the protein expression profiles in the testosterone group with the control group, and testosterone group + PWE with the testosterone group, both down-expressed and overexpressed proteins were highlighted (Figures 6H,I).

In particular, several proteins such as *Cytochrome c*, *Ubiquitin carboxyl-terminal hydrolase isozyme L1* (UCH-L1), *Thioredoxin domain-containing protein 12*, were down-expressed in testosterone group respect to control group. Such proteins may act as tumor suppressors counteracting oxidative stress (Figure 6H).

Evaluating the effect induced by PWE in the testosterone group we have highlighted that PWE treatment is able to counteract the effect of modulating the expression of *Cytochrome c*, *Ubiquitin carboxyl-terminal hydrolase isozyme L1* (UCH-L1), *Thioredoxin domain-containing protein 12* proteins, bringing the values back to those of the control. Moreover, PWE treatment in testosterone group, induced over-expression of *Superoxide dismutase (SOD)*, *Histone H4* and *ATP synthase Subunit d*, proteins that assisting in DNA repair during DNA replication and acting as superoxide anion scavenger in the prostate can reduce oxidative stress caused by testosterone (Figure 6I).

Data obtained in our experimental conditions highlighted also that *Glutathione S-transferases* (GSTs), and *Tumor-associated calcium signal transducer 2* (TROP-2) were down-expressed in testosterone group + PWE respect to testosterone group. These results suggest that in testosterone group the expression of GSTs can represent an adaptive response of the prostate to testosterone, while TROP-2 expression could serve as a potential prognostic biomarker acting as intracellular calcium signal transducer linked to processes like self-renewal, proliferation and invasion. The treatment with PWE was able to reduce the expression of these proteins highlighting its ability to counteract the effect of testosterone and to decrease prostate cells proliferation (Figure 6I).

The heatmap summarizes how testosterone alone and T + PWE modulate the abundance of multiple proteins involved in cytoskeletal organization, metabolism, redox balance, and stress responses relative to control (Figure 6J). Many proteins are clearly upregulated by testosterone, as indicated by the intense red color in the T column, whereas the corresponding T + PWE values are often lighter, suggesting that the extract blunts several testosterone-driven changes. The heatmap also includes proteins below significance threshold to illustrate global patterns.

### 3.5 Oxidative stress and inflammation in rat PPAT

Periprostatic adipose tissue (PPAT), the fat surrounding the prostate, is increasingly recognized as an active endocrine and paracrine organ that influences BPH. This fat can release factors like hormones and cytokines that affect prostate reactivity and growth, potentially promoting BPH development and worsening urinary symptoms.

PPAT influences BPH releasing inflammatory mediators, including cytokines and hormones, that can increase prostate tissue’s growth and reactivity, contributing to BPH development. PPAT produce adipokines, such as leptin and adiponectin, and other signaling proteins that can affect various physiological processes in the prostate and that play a role in inflammation and tissue growth (Fibbi et al., 2010; Sacca and Calvo, 2022).

**FIGURE 7:**
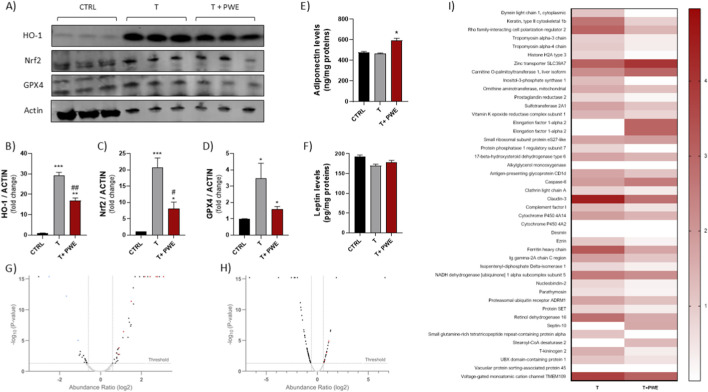
**(A–D)** Representative images and quantification of HO-1, Nrf2 and GPX4 expression in PPAT following treatment by Western blot. **(E,F)** Assessment of Adiponectin and Leptin levels, respectively, in PPAT. **(G,H)** Volcano plot depicting differentially expressed proteins in hepatic tissue from respectively testosterone-treated (T) vs. control (Ctrl) groups and (T + PWE) vs. (T). Each point represents a protein, with x-axis indicating log_2_ abundance ratio and y-axis showing -log_10_ adjusted p-value. Horizontal dashed line denotes significance threshold (adj. p < 0.05); vertical dashed lines mark fold-change thresholds (±1.5). **(I)** Representative heatmap of differentially expressed proteins in liver tissue following treatments. Heatmap illustrating the abundance ratios (0–4) of proteins modulated in rat hepatic tissue after treatment with T or T + PWE. Rows correspond to individual proteins identified by LC-MS/MS analysis, and columns represent the treatment conditions. Color intensity reflects normalized protein abundance, with darker shades indicating higher expression levels.

In the PPAT, a distinct redox-related response was observed (Figures 7A–D) following hormonal and phytochemical treatments. Specifically, testosterone administration led to a significant upregulation of HO-1, NRF2, and GPX4 expression, indicating the activation of adaptive antioxidant and cytoprotective mechanisms within the adipose microenvironment. Conversely, co-treatment with PWE markedly reduced the expression levels of all three markers compared to testosterone alone, restoring them toward basal or control-like values. These findings suggest that PWE counteracts testosterone-induced redox imbalance in PPAT.

Measurement of adipokines as adiponectin and leptin in PPAT samples showed that PWE co-treatment was able to increase adiponectin levels in PPAT compared to testosterone alone, indicating a shift toward a more anti-inflammatory and metabolically favorable adipose profile. In the context of androgen exposure, where PPAT typically acquires a pro-inflammatory secretory pattern, the upregulation of adiponectin suggests that PWE can partially restore protective adipokine signaling within PPAT tissue samples (Figure 7E). This effect is particularly relevant given adiponectin’s known ability to counteract cytokine-driven inflammation, improve insulin sensitivity, and modulate tissue remodeling, implying that higher adiponectin in PPAT may attenuate paracrine cues that promote prostatic growth and reactivity (Choi et al., 2020; Karnati et al., 2017). On the other hand, no significant alterations were observed in leptin levels analyzed in PPAT samples (Figure 7F).

### 3.6 Liver tissue proteomic analysis

To investigate whether testosterone, although not eliciting an increase in prostate volume, induced metabolic alterations at the hepatic level, a proteomic analysis was conducted. Results from volcano plot analysis showed that comparing the protein expression profiles in the testosterone group with the control group, down-expressed and overexpressed proteins were highlighted (Figures 7G,H).

In particular, *Glutathione S-transferases* (GSTs), *Mitochondrial ATP synthase* and *Cytochrome c oxidase* were down-expressed in testosterone group respect to control group. These results highlight that dysregulation of GSTs can lead to the accumulation of toxic metabolites, DNA damage and increased inflammation, as well as of *Mitochondrial ATP synthase* and *Cytochrome c oxidase*, are a consequence of oxidative stress (Figure 7G).

Several proteins such as, *Cytochrome P450 A14* (CYP4A14), *Cytochrome P450 A2*, or CYP2A, *17-β-hydroxysteroid dehydrogenase type 6* (HSD17B6), *Retinol dehydrogenases* (RoDHs), *Carnitine palmitoyltransferase* (CPT), *Elongation factor 1 alpha* (EF-1α), *Alkylglycerol monooxygenase* (AGMO), *claudin-3*, *Stearoyl-CoA desaturase* (SCD), *Caspase-6* were overexpressed in testosterone group respect to control group. These results highlight that CYP4A14 overexpression may be related to liver lipid accumulation and inflammation, while CYP2A enzymes in rats can catalyze testosterone 7α-hydroxylation (Figure 7G). It has been reported that enzymes such as liver HSD17B6, RoDHs, claudin-3 and EF-1α are induced by testosterone because they are involved in the regulation of androgen metabolism while Caspase-6 plays a critical role in liver injury promoting inflammation and cell death (Schiffer et al., 2018).

Results from volcano plot analysis showed that comparing the protein expression profiles in the T + PWE group with the testosterone group, overexpressed proteins are highlighted (Figure 7H). In particular, *Mitochondrial ATP synthase*, *Thioredoxin*, *glutathione S-transferase* (GST) and glutathione peroxidase (GPx) were overexpressed highlighting that PWE treatment was able to induce synthesis of proteins involved in maintaining mitochondrial membrane potential, in detoxification of harmful substances, in protection of cells from oxidative damage. These enzymes are part of the liver’s antioxidant defense system and reducing peroxides they promote regeneration of glutathione (GSH) to maintain cellular health (Asantewaa et al., 2024).

In liver, testosterone also induced a coordinated proteomic response, characterized by increased expression of cytoskeletal and polarization markers, lipid and xenobiotic-metabolizing enzymes, and proteins involved in translation, protein turnover, immune function, and apoptosis (Figure 7I). PWE co-treatment attenuated many of these testosterone-dependent changes, although several metabolic and signaling proteins remained elevated or were further increased, indicating that PWE potentially modulates rather than uniformly suppresses the hepatic response to androgen stimulation. The heatmap also includes proteins below significance threshold to illustrate global patterns.

## 4 Discussion

Benign prostatic hyperplasia (BPH) is a common age-related condition characterized by the non-malignant enlargement of the prostate gland, often driven by androgenic stimulation and accompanied by complex molecular, cellular, and tissue-level alterations. Oxidative stress, inflammation, and microvascular dysfunction are recognized key contributors to BPH pathogenesis, creating an environment conducive to structural remodeling and impaired tissue homeostasis. Natural compounds with antioxidant and anti-inflammatory properties have emerged as promising adjuncts to mitigate these changes, potentially complementing conventional therapies. This study investigates the histological, molecular, proteomic, and redox effects of testosterone-induced prostatic alterations and explores how co-treatment with pomegranate waste extract (PWE), rich in bioactive phytochemicals, modulates these processes across prostate tissue, periprostatic adipose tissue (PPAT), and liver.

Histological analysis of ventral prostate sections reveals significant morphological changes induced by testosterone and modified by co-treatment with pomegranate waste extract (PWE). Prostates from control animals exhibit well-organized glandular architecture, characterized by uniform acini with a single epithelial cell layer, either cuboidal/columnar or pseudostratified columnar, consistent with normal rodent prostate histology (Ginja et al., 2019). Basally localized nuclei and intact cytoplasm further confirm tissue homeostasis under physiological conditions.

Testosterone administration markedly alters this morphology, aligning with previous reports of androgen-driven epithelial hyperplasia and early benign prostatic hyperplasia (BPH) remodeling (Li et al., 2018). Specifically, columnar epithelial cells with basal nuclei and perinuclear cytoplasmic clearing indicate heightened secretory activity and cellular stress linked to androgen overstimulation (Kayode et al., 2020). Villous epithelial projections and pseudostratified arrangements suggest increased epithelial turnover and glandular infolding, hallmarks of testosterone-induced prostatic enlargement models (Lovett et al., 2017). Interestingly, PWE co-treatment retains these features but intensifies villous projections, possibly reflecting phytochemical-driven modulation of epithelial dynamics and stromal interactions. Natural compounds exhibiting antioxidant and anti-inflammatory properties can influence hormone-dependent epithelial morphology by mitigating oxidative stress or regulating differentiation pathways (Yeewa et al., 2020). Thus, the observed epithelial alterations may represent an adaptive response to combined hormonal and phytochemical stimuli. These histological findings validate the hypothesis that testosterone induces structural remodeling of the ventral prostate, while PWE exerts measurable modulatory effects at the tissue level.

At the molecular level, testosterone provokes redox imbalances and endothelial dysfunction in prostate tissue, consistent with oxidative stress, inflammation, and vascular changes implicated in BPH pathogenesis. Testosterone significantly reduces PECAM-1 expression, a vital adhesion molecule maintaining endothelial integrity, angiogenesis, and leukocyte trafficking; its downregulation correlates with microvascular dysfunction and tissue remodeling (Eid and Abdel-Naim, 2020; Newman and Newman, 2003). Concurrently, antioxidant defense mechanisms, notably NRF2 and HO-1, are upregulated following testosterone exposure, likely as compensatory responses to increased reactive oxygen species (ROS) (Jahan et al., 2021). However, persistent oxidative stress and endothelial injury may promote pathological remodeling despite this acute activation. Remarkably, PWE co-treatment normalizes PECAM-1, NRF2, and HO-1 levels toward control values, suggesting restoration of endothelial function and redox homeostasis, an effect consistent with polyphenols’ antioxidant and anti-inflammatory actions in testosterone-induced BPH (Csikós et al., 2021; Park et al., 2022). Inflammatory markers corroborate these molecular shifts: testosterone elevates IL1R1 expression, indicative of pro-inflammatory signaling, while suppressing anti-inflammatory IL-10. PWE maintains IL-10 expression at control levels, preserving anti-inflammatory balance and illustrating its immunomodulatory capacity to prevent chronic inflammation central to BPH progression (Akdere et al., 2025). Redox biomarker evaluation reveals that while total thiols (RSH/GSH) remain stable, lipid hydroperoxides (LOOH) decrease slightly with PWE treatment, reflecting reduced lipid peroxidation and membrane damage. Given the prostate’s vulnerability to oxidative injury, this attenuation aligns with previous findings that antioxidant phytochemicals mitigate lipid peroxidation in BPH models (Kaltsas et al., 2025; Arias-Chávez et al., 2023; Consoli et al., 2023). Overall, these observations indicate that PWE counteracts androgen-mediated prostatic injury by sustaining endothelial homeostasis, limiting oxidative stress, and modulating inflammatory responses. By normalizing PECAM-1, NRF2, HO-1, IL1R1, IL-10, and reducing lipid peroxidation, PWE supports tissue homeostasis and may slow structural remodeling underlying BPH. These findings endorse the potential of nutraceutical strategies as adjunctive therapies to conventional BPH treatments (Cicero et al., 2019; Stewart and Lephart, 2023).

To further substantiate testosterone’s impact on oxidative stress and inflammation, proteomic profiling was performed. Volcano plot analysis revealed that testosterone markedly disrupts proteins related to mitochondrial function, redox regulation, and cellular homeostasis. Key mitochondrial and antioxidant molecules such as Cytochrome c, UCH-L1, and thioredoxin domain-containing protein 12 were significantly downregulated. Decreased Cytochrome c aligns with evidence that androgens impair mitochondrial electron transport, increase ROS, and contribute to hyperplasia and tissue remodeling (Kimura et al., 2001). UCH-L1 downregulation is notable due to its tumor-suppressive, genome-stabilizing role and association with resistance to oxidative damage. Similarly, reduced thioredoxin-related proteins indicate diminished antioxidant defense capacity, heightening vulnerability to testosterone-induced oxidative injury (Lu and Holmgren, 2014). Importantly, PWE co-treatment largely restored Cytochrome c, UCH-L1, and thioredoxin protein levels near baseline, supporting pomegranate phytochemicals’ capacity to alleviate mitochondrial dysfunction and oxidative imbalance. This aligns with reports of polyphenol-rich extracts reducing mitochondrial ROS generation and enhancing redox enzymes in androgen-driven prostatic damage (Bakalova et al., 2022; Cerqueira et al., 2020). Furthermore, PWE significantly upregulated Superoxide Dismutase (SOD), a primary ROS-detoxifying enzyme, consistent with antioxidant phytochemical activities documented in BPH redox models (Kucukdurmaz et al., 2017; Sreekumar et al., 2023).

Additional proteomic changes underscore PWE’s protective effects: glutathione S-transferases (GSTs) and TROP-2 expression were reduced in the testosterone + PWE group relative to testosterone alone. Testosterone-induced GST upregulation likely reflects an adaptive response to oxidative and electrophilic stress (Hayes and Pulford, 1995), while PWE-induced normalization suggests diminished oxidative load and detoxification demand. TROP-2, a calcium signal transducer implicated in proliferation, stemness, and tumor aggressiveness (Trerotola et al., 2013), was downregulated by PWE, indicating attenuation of pro-proliferative signaling. Given TROP-2’s role in epithelial turnover and tumor progression, its reduction highlights PWE’s potential to limit pathological microenvironmental cues. Broadly, testosterone induced upregulation of proteins involved in cytoskeletal organization, mitochondrial metabolism, redox/detoxification systems, and stress responses. PWE co-treatment generally diminished these increases towards control levels, suggesting normalization of androgen-driven structural and metabolic remodeling. Notably, testosterone promoted cytoskeletal and contractility-related proteins associated with reinforced actin–microtubule architecture, indicative of enhanced cell motility and remodeling. PWE reduced these cytoskeletal protein signals, possibly restoring a more quiescent cellular phenotype. Similarly, testosterone-enhanced mitochondrial enzymes implied increased energetic and oxidative demand, which PWE mitigated, consistent with its antioxidant activity. Indeed, androgen exposure, even in the absence of overt prostatic hypertrophy, elicits a coordinated pathogenic programme involving oxidative stress, endothelial dysfunction, inflammatory activation and proteomic remodeling in the ventral prostate, periprostatic adipose tissue and liver. Co-administration of pomegranate waste extract (PWE) consistently attenuated these alterations, restoring endothelial and redox markers (PECAM-1, NRF2, HO-1) towards basal levels, preserving anti-inflammatory signaling (IL-10), limiting testosterone-induced changes in mitochondrial and cytoskeletal proteins and reinforcing antioxidant defences in both prostate and liver.

Periprostatic adipose tissue (PPAT) functions as an active endocrine and paracrine compartment influencing prostate homeostasis and BPH development by secreting adipokines and cytokines that modulate inflammation, smooth muscle tone, and epithelial proliferation (Cao et al., 2024; Passos et al., 2021). Testosterone triggered pronounced redox responses in PPAT, increasing expression of HO-1, NRF2, and GPX4, key antioxidant and lipid peroxidation regulators, reflecting adaptive oxidative stress responses that may alter PPAT secretory profiles and paracrine effects on the prostate. PWE co-treatment attenuated these increases, restoring levels near baseline, suggesting phytochemical modulation of PPAT redox status could help reduce pro-inflammatory and growth-promoting signals contributing to BPH. Furthermore, PWE not only acts directly on prostate tissue but also seems to be able to beneficially reprogram the surrounding adipose microenvironment, potentially contributing to the overall attenuation of BPH-related changes. Proteomic analysis of liver tissue revealed that testosterone induces metabolic remodeling, even without overt prostate enlargement. Downregulation of detoxifying and mitochondrial proteins indicates compromised redox homeostasis and oxidative phosphorylation, predisposing the liver to toxic metabolite accumulation, oxidative damage, and inflammation (Staffas et al., 1998). Conversely, testosterone upregulated enzymes associated with xenobiotic metabolism, androgen processing, lipid metabolism, and apoptosis. These changes collectively suggest enhanced metabolic stress, lipid accumulation, inflammatory signaling, and hepatocyte injury. PWE alleviated these alterations by boosting detoxification and peroxide clearance systems, supporting hepatic redox balance in line with pomegranate extract’s hepatoprotective effects (Ali et al., 2021). Overall, the liver proteomic heatmap illustrates testosterone-induced hepatic remodeling involving cytoskeletal organization, lipid/xenobiotic metabolism, protein turnover, redox/iron handling, and immune-apoptotic pathways. PWE co-treatment partially attenuated these androgen-driven proteomic changes, modulating but not fully reversing them, suggesting a tissue-specific reshaping rather than complete suppression of testosterone’s hepatic effects. Therefore, our findings support a multifaceted protective role of PWE against androgen-induced alterations in prostate, PPAT, and liver, acting on endothelial integrity, redox balance, inflammatory signalling, and proteomic remodeling. This reinforces the concept that polyphenol-rich nutraceuticals may complement standard BPH therapies by targeting systemic and microenvironmental drivers of disease progression (Cicero et al., 2019; Stewart and Lephart, 2023).

## 5 Conclusion

Collectively, the data herein reported identify PWE as a candidate nutraceutical adjunct capable of constraining androgen-driven tissue injury and microenvironmental dysregulation that contribute to BPH pathogenesis. Future work should address the long-term impact of PWE on prostate volume, lower urinary tract function and structural remodeling end points, including fibrosis and stromal expansion, and should systematically delineate the relevant molecular pathways, such as NF-κB signaling, apoptotic circuits and profibrotic cascades. From a translational perspective, integration of PWE or related polyphenol-rich formulations into preventive or early-intervention strategies, alone or in combination with standard pharmacotherapies, warrants investigation to define efficacy, safety, optimal dosing and patient subsets most likely to benefit.

## Data Availability

The original contributions presented in the study are publicly available. This data can be found here: ProteomeXchange Consortium via the MassIVE repository, accession number PXD074386. Additionally you can find the related link as follows: https://proteomecentral.proteomexchange.org/cgi/GetDataset?ID=PXD074386.
